# Antimicrobial steroidal saponin and oleanane-type triterpenoid saponins from *Paullinia pinnata*

**DOI:** 10.1186/1472-6882-14-369

**Published:** 2014-10-02

**Authors:** Paul K Lunga, Xu-Jie Qin, Xing W Yang, Jules-Roger Kuiate, Zhi Z Du, Donatien Gatsing

**Affiliations:** Department of Biochemistry, Laboratory of Microbiology and Antimicrobial Substances, Faculty of Science, University of Dschang, P.O. Box 67, Dschang, Cameroon; State Key Laboratory of Phytochemistry and Plant Resources in West China, Kunming Institute of Botany, Chinese Academy of Science, Kunming, 650201 P R China; Department of Biochemistry, Laboratory of Phytobiochemistry and Medicinal Plants Study, Faculty of Science, University of Yaoundé 1, P.O. Box 812, Yaoundé, Cameroon

**Keywords:** *Paullinia pinnata*, Sapindaceae, Steroidal saponin, Oleanane triterpenoid saponins, Antimicrobial activities

## Abstract

**Background:**

*Paullinia pinnata* L. (Sapindaceae) is an African woody vine, which is widely used in traditional medicine for the treatment of human malaria, erectile dysfunction and bacterial infections. A phytochemical investigation of its methanol leaf and stem extracts led to the isolation of seven compounds which were evaluated for their antimicrobial properties.

**Methods:**

The extracts were fractionated and compounds were isolated by chromatographic methods. Their structures were elucidated from their spectroscopic data in conjunction with those reported in literature. The antimicrobial activities of the crude extracts, fractions and compounds were evaluated against bacteria, yeasts and dermatophytes using the broth micro-dilution technique.

**Results:**

Seven compounds: 2-*O*-methyl-L-chiro-inositol (**1**), *β*-sitosterol (**2**), friedelin (**3**), 3*β*-(*β*-D-Glucopyranosyloxy) stigmast-5-ene (**4**), (3*β*)-3-*O*-(2′-Acetamido-2′-deoxy-*β*-D-glucopyranosyl) oleanolic acid (**5**), (3*β*,16*α-*hydroxy)-3-*O*-(2′-Acetamido-2′-deoxy-*β*-D-glucopyranosyl) echinocystic acid (**6**) and (3*β*)-3-*O*-[*β*-D-glucopyranosyl-(1″-3′)-2′-acetamido-2′-deoxy-*β*-D-galactopyranosyl]oleanolic acid (**7**) were isolated. Compounds **5** and **7** showed the best antibacterial and anti-yeast activities respectively (MIC value range of 0.78-6.25 and 1.56-6.25 μg/ml), while **6** exhibited the best anti-dermatophytic activity (MIC value range of 6.25-25 μg/ml).

**Conclusion:**

The results of the present findings could be considered interesting, taking into account the global disease burden of these susceptible microorganisms, in conjunction with the search for alternative and complementary medicines.

## Background

*Paullinia pinnata* L. (Sapindaceae), an African woody vine, whose leaves and roots are widely used in traditional medicine for the treatment of human malaria [1] and erectile dysfunction [2]. In the West Region of Cameroon, its leaf decoction is used for the treatment of bacterial infections like typhoid fever, syphilis, gonorrhea, diarrhea, and symptoms like stomach-ache and waist pain. In East Africa, the leaves are reported to be used in the treatment of gonorrhea, wounds and microbial infections [3]. Previous phytochemical investigations have shown the presence of triterpene saponins, cardiotonic catechol tannins [4, 5], flavone glycosides [6], steroids, steroidal glycosides [7], cerebroside and ceramide [8], as well as fatty acids [1] in *P. pinnata* collected from different parts of Africa.

There is a very limited biological investigation of the chemical constituents from the title species *P. pinnata.* As a result of our interest in the chemical and biological investigation of this plant, a methylinositol (**1**), steroidal terpenoids (**2** and **4**), and oleanane triterpenoids (**3**, **5**–**7**) were isolated from its leaf and stem methanol extracts. In our previous studies, the antityphoid and antioxidant properties of these compounds were evaluated [9]. The extracts, fractions and all the compounds were evaluated for their antimicrobial activities against eight bacteria, five yeasts and five dermatophytes species and the results are reported herein. Compounds **2** (*β*-sitosterol) and **4** (daucosterol) were formally isolated from *P. pinnata* leaves collected from Cameroon [7]. To the best of our knowledge, **1**, **3** and **5**–**7** are isolated from *P. pinnata* for the first time, and the antimicrobial properties of **5**–**7** are presented here for the first time.

## Methods

### Plant material

The air-dried leaves and stems of *P. pinnata* were obtained from Dschang, West Region of Cameroon, in January 2009. The identification of plant specimens was done at the Cameroon National Herbarium in Yaounde by Mr Tadjouteu Fulbert, where a voucher specimen was deposited under the reference number 10702/SRFCam.

### Extraction and isolation

The air-dried leaves (2.04 Kg) and stems (2.02 kg) of *P. pinnata* were powdered and extracted with MeOH (7 l × 2, 48 h each) at room temperature to give crude extracts (233.8 g and 152.17 g respectively) after concentration under reduced pressure. The leaf extract (230 g) was exhaustively and successively washed with n-hexane and acetone to afford the hexane (45.2 g), acetone (8 g) and methanol residue (156.8 g) fractions while the stem extract was partitioned into petroleum ether, ethyl acetate and water to obtain the PE fraction (8.08 g), EtOAc fraction (9.13 g) and aqueous residue fraction (109.89 g).

One hundred and fifty grams of the methanol residue fraction was applied to neutral silica gel 60 (0.2-0.5 mm) column (60 × 8 cm) and eluted with mixtures of n-hexane-ethyl acetate and ethyl acetate-methanol of increasing polarity (100:0 → 0:100 with constant polarity increase of 5%). This gave 60 fractions which were grouped on the basis of their TLC band pattern similarities into 5 fractions (F1 to F5). Further column purification of F2 (eluted with EtOAc-MeOH, 90:10) on silica gel yielded six fractions denoted F2.1 to F2.6. Niddle-like crystals, formed in F2.3 (EtOAc-MeOH, 95:5) and F2.4 (EtOAc-MeOH, 90:10) were collected and purified on a sephadex gel (LH-20), eluted with an isocratic system of CHCl_3_-MeOH (40:60) to afford 2-*O*-methyl-L-chiro-inositol (**1**, 28 mg). Fourty grams of the hexane fraction was applied to neutral silica gel 60 (0.2-0.5 mm) column (60 × 8 cm) and eluted with a mixture of petroleum ether-ethyl acetate of increasing polarity (100:0 → 50:50 with constant polarity increase of 5%) to give 40 fractions. These fractions were further grouped on the basis of their TLC band pattern similarities into 5 fractions (F1 to F5). Fraction F1 (Petroleum ether 100%) was mounted on a silica gel column and eluted with a mixture of Hex-EtOAc of increasing polarity (95:5 → 50:50) to yield 30 fractions which were equally grouped on the basis of their TLC band pattern similarities into 5 sub fractions (F1.1 to F1.5). Sub fractions F1.1 (Hex-EtOAc, 90:10) and F1.4 (Hex-EtOAc, 65:35) both yielded white powders which were purified by sephadex gel (LH-20) column chromatography and eluted with CHCl_3_-MeOH (4:6) to afford *β*-sitosterol (**2**, 20 mg). Finally, F2 (Petroleum ether-EtOAc, 95:5) was mounted on a silica gel column and eluted with a mixture of Hex-EtOAc of increasing polarity (95:5 → 70:30) to yield 10 fractions which were grouped on the basis of their TLC band pattern similarities into 3 sub fractions (F2.1 to F2.3). Sub fractions F2.1 (Hex-EtOAc, 95:5) and F2.2 (Hex-EtOAc, 93:7) yielded transparent crystals which were purified by sephadex gel (LH-20) column chromatography and eluted with CHCl_3_-MeOH (4:6) to affordfriedelin (**3**, 18 mg).

The EtOAc fraction (7.07 g) was subjected to column chromatography on Rp-18 gel (MPLC, MeOH-H_2_O 50:50 → 100:0) to afford 3*β*-(*β*-D-Glucopyranosyloxy) stigmast-5-ene (**4**, 119 mg), (3*β*)-3-*O*-(2′-Acetamido-2′-deoxy-*β*-D-glucopyranosyl) oleanolic acid (**5**, 170 mg) and 8 fractions. Similarly, F4 (3.60 g) was chromatographed on silica gel column and eluted with CHCl_3_-MeOH (9:1 → 7:3) to give 4 fractions. F4.4 (448 mg) was subjected to sephadex LH-20 gel column chromatography and eluted with CHCl_3_-MeOH (1:1) to afford (3*β*, 16*α-*hydroxy)-3-*O*-(2′-Acetamido-2′-deoxy-*β*-D-glucopyranosyl) echinocystic acid (**6**, 45 mg). The aqueous residue fraction (76.79 g) was mounted on a D101 macroporous resin column and eluted successively with H_2_O-EtOH (10:0; 7:3; 5:5; 3:7; 0:10) to obtain 5 fractions denoted F1 to F5 respectively. F5 (946 mg) was purified on a silica gel column, eluted with a stepwise gradient mixture of CHCl_3_-MeOH-H_2_O (8:2:0.5 → 6:4:0.5) to afford (3*β*)-3-*O*-[*β*-D-glucopyranosyl-(1″-3′)-2′-acetamido-2′-deoxy-*β*-D-galactopyranosyl] oleanolic acid (**7**, 40 mg).

### Identification of compounds

Optical rotations were measured with a JASCO P-1020 digital polarimeter. UV spectra were obtained using a Shimadzu UV-2401 PC spectrophotometer. IR spectra were recorded on a Bruker Tensor-27 infrared spectrophotometer using KBr pellets. 1 day and 2D NMR spectra were performed on Bruker AM-400 and DRX-500 spectrometers (BrukerBioSpinGmBH, Rheinstetten, Germany) with TMS as the internal standard. ESIMS spectra were recorded on a Bruker HTC/Esquire spectrometer. HREIMS was recorded on a Waters AutoSpec Premier P776 spectrometer. Column Chromatography (CC) was performed on silica gel (200–300 mesh, Qingdao Marine Chemical Ltd., Qingdao, China), Rp-18 (40–63 μm, Merk). Fractions were monitored by TLC (GF254, Qingdao Marine Chemical Ltd., Qingdao, China), and by heating silica gel plates sprayed with 10% H_2_SO_4_ in ethanol. GC analysis was performed on an HP5890 gas chromatograph equipped with a H_2_ flame ionization detector.

### Acidic hydrolysis of 4–7, and GC analysis

Compounds **4**-**7** (2 mg) were each refluxed with 2 M HCl (1,4-dioxane/H_2_O 1:1, 2 ml) on water bath for 2 h. After cooling, the reaction mixture was extracted with CHCl_3_ (3 × 5 ml). The aqueous layer was evaporated to dryness with MeOH until neutral. The dried residue (sugar) was dissolved in 1 ml anhydrous pyridine and treated with L-cysteine methyl ester hydrochloride (1.5 mg), and stirred at 60°C for 1 h. Trimethylsilylimidazole (1.0 ml) was added to the reaction mixture, and this was kept at 60°C for 30 min. The resulting supernatant (4 μl) was analyzed by GC under the following conditions: H_2_ flame ionization detector; Column: 30QC2/AC-5 quartz capillary column (30 m × 0.32 mm); Column temperature: 180–280°C at the rate of 3°C/min;carrier gas: N_2_ (1 ml/min); injector temperature: 250°C; split ratio: 1/50. The configurations of D-glucose and D-galactose for compounds **4**–**7** were determined by comparison of the retention times of their corresponding derivatives with those of standard D-glucose and D-galactose giving a single peak at 10.669 and 10.969 min, respectively.

### Antimicrobial assays

#### Microorganisms and culture media

The microorganisms used in this study were obtained from the American Type Culture Collection (ATCC), “EcoleNationaleVétérinaired’Alfort” (E), “centre Pasteur” of Yaounde-Cameroon and “Institut Pasteur” of Paris-France (IP). They includedeight bacteria strains: *Salmonella typhi* ATCC 6539, *Staphylococcus aureus* ATCC 25922, *Pseudomonas aeruginosa* ATCC 27853, *Klebsiella pneumoniae* ATCC 13883, *Escherichia coli* ATCC 10536, *Enterococcus faecalis* ATCC 10541, *Enterobacter aeroginese* ATCC 13048, *Providensia smartii* ATCC 29916; five yeasts: *Candida albicans* ATCC 2091, *Candida guiliermondii*, *Cryptococcus neoformans* IP 90526, *Candida luciteniae* ATCC 200950 and *Candida parapsilosis* ATCC 22019 and five dermatophytes: *Trichophyton equinum* E1424, *Microsporium audouinii* E1421, *Trichophyton mentagrophytes* E1425, *Microsporium gypseum* E1420 and *Epidermophyton flocosum.*

The culture media, Nutrient Agar (NA, Conda) and Sabouraud Dextrose Agar (SDA, Conda), were used for culturing bacteria and fungi respectively, while Mueller Hinton Broth (MHB, Conda), and Sabouraud Dextrose Broth (SDB, Conda) were used for the determination of minimum inhibitory and minimum microbicidal concentrations.

#### Preparation of microbial inocula

The inocula of bacteria and yeasts were prepared from 24 h and 48 h old agar cultures respectively. The absorbance was read at 600 nm (Jenway 6105 UV/Vis spectrophotometer- 50 Hz/60 Hz) and adjusted with sterile physiological solution to match that of a 0.5 McFarland standard solution. From the prepared microbial solutions, other dilutions with sterile physiological solution were prepared to give a final concentration of 10^6^ colony- forming units (CFU) per milliliter for bacteria and 2 × 10^5^ spores per milliliter for yeasts [10].

Conidia suspensions of dermatophyte species were prepared from 10 days old cultures. The number of conidia was determined using a spectrophotometer and adjusted with sterile saline (NaCl) solution (0.9%) to an absorbance of 0.600 at 450 nm,corresponding to a final concentration of about 1 × 10^5^ spores/ml [11].

#### Determination of minimum inhibitory concentration (MIC) and minimum microbicidal concentration (MMC)

The MICs of the samples were determined by the broth microdilution method in 96-well micro-titre plates as described in the literature elsewhere [12]. The 96-well plates were prepared by dispensing into each well 100 μl of Mueller Hinton broth for bacteria and Sabouraud Dextrose broth for fungi. The test substances were initially prepared in 10% DMSO in broth medium and 100 μl of each test sample was added into the first wells of the micro-titre plate (whose wells were previously loaded with 100 μl of broth medium). Serial two-fold dilutions of the test samples were made and 100 μl of inoculum were then added into each well. This gave final concentration ranges of 12500 to 6.10 μg/ml for extract and fractions, 100 to 0.78 μg/ml for the compounds and 12.5 to 0.09 μg/ml for reference substances. The plates were sealed with parafilm, then agitated with a plate shaker to mix their contents and incubated at 35°C for 24 h for bacteria, 48 h for yeast and at 28°C for 7 days for dermatophytes.

For bacteria, MICs were determined upon addition of 50 μl (0.2 mg/ml) p-iodonitrotetrazolium chloride (INT, Sigma-Aldrich, South Africa). Viable bacteria reduced the yellow dye to a pink color. For yeasts and dermatophytes, MICs were determined by visualizing the turbidity of the wells. The MIC corresponded to the lowest well concentration where no color/turbidity change was observed, indicating no growth of microorganism. All tests were performed in triplicates.

Minimum microbicidal concentrations were determined by adding 50 μl (for bacteria and yeasts) or 5 μl (for dermatophytes) aliquots of the preparations (without INT for bacteria), which did not show any visible color/turbidity change after incubation during MIC assays, into 150 or 195 μl of sample-free broth. These preparations were further incubated and revealed as above to obtain the MMCs. Gentamycin, nystatin and griseofulvin were used as positive controls for bacteria, yeast and dermatophytes respectively.

## Results and discussion

**Figure 1:**
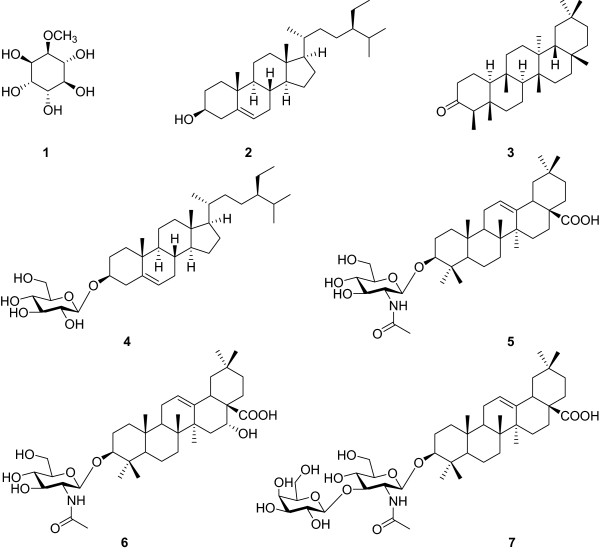
**Chemical structures of compounds from the leaves (1–3) and stems (4–7) of** ***P. pinnata.***

The following known compounds: 2-*O*-methyl-L-chiro-inositol (**1**) [13] whose ^13^C NMR data were very close to those of L-quebrachitol [14, 15], *β*-sitosterol (**2**) [16], friedelin (**3**) [17, 18] (Figure 1) were isolated and identified in the leaves of *P. pinnata*. From the MeOH stem- extract, 3*β*-(*β*-D-Glucopyranosyloxy) stigmast-5-ene or daucosterol (**4**) [19], 3-*O*-(2′-acetamido-2′-deoxy-*β*-D-glucopyranosyl) olean-12-en-28-oic acid or aridanin (**5**) [20, 21], 3-*O*-(2-acetamido-2′deoxy-*β*-D-glucopyranosyl)-l6*α*-hydroxyolean-l2-en-28-oic acid (**6**) [20] and 3-*O*-[*β*-D-glucopyranosyl-(1″-3′)-2′-acetamido-2′-deoxy-*β*-D-galactopyranosyl] olean-12-en-28-oic acid or lotoidoside E (**7**) [22] were isolated and identified (Figure 1). The compounds isolated in the present study were formerly isolated from other plants and the biological activities of some were demonstrated [23, 24]. Besides, in our previous investigation, their antityphoid and antioxidant properties were demonstrated [9]. *P. pinnata* extract have been proven to possess antioxidant [25] and vascular relaxation [2] properties.

The antimicrobial properties of the extracts, fractions and isolated compounds of the leaves and stems of *P. pinnata* are presented in Tables 1 and 2 respectively. In general, the crude extract of the leaves presented a better antibacterial activity compared to the stem extract; both of whose activities were comparable and not significant on fungi especially on dermatophytes. The results show that Compound **5** and **7** exhibited significant antibacterial and anti-yeast activities respectively (MIC value range of 0.78-6.25 and 1.56-6.25 μg/ml), while **6** exhibited the best anti-dermatophytic activity (MIC value range of 6.25-25 μg/ml). **1** was the most active antibacterial compound from the leaves, but was less active compared to **5**. No compound from the leaves exhibited antifungal activity at the tested concentration. This probably explains why *P. pinnata* is not used by the local population in the treatment of fungal infections, since the leaf is the part locally used. *β*-sitosterol (**2**) was formally isolated from *Citrus grandis* fruits and shown to possess activity against gram-positive (*Bacillus cereus, Bacillus subtilis* and *Staphylococcus aureus*) and gram-negative (*Escherichia coli* and *Salmonella enteritidis*) bacteria, with MIC value of 300 μg/ml [26]. Friedelin (**3**), isolated from the stem bark of *Vismia rubescens* demonstrated antibacterial activity against *Salmonella typhi, Staphylococcus aureus, Pseudomonas aeruginosa* with MIC values of 25–200 μg/ml [27]. Besides, daucosterol (4) is a known antibacterial compound [28]

**Table 1:** **Minimum inhibitory and cidal concentrations (μg/ml) of the extract, fractions and compounds from the leaves of** ***P. pinnata*** **against bacteria and fungi**

Microorganism		Extract ^a^	H _f_	A _f_	MR _f_	1	2	3	RD
**Bacteria**									
*S. aureus*	MIC	390	97	195	48	50	/	/	0.781
	MBC	390	390	390	97	/	/	/	3.125
*P. aeruginosa*	MIC	390	195	781	781	6.25	/	100	0.781
	MBC	1562	781	1562	1562	50	/	100	1.562
*K. pneumonia*	MIC	195	390	97	781	12.5	/	50	6.25
	MBC	390	1562	781	1562	100	/	100	6.25
*E. coli*	MIC	48	24	12	12	0.781	100	50	0.781
	MBC	195	97	48	24	12.5	/	50	0.781
*E. faecalis*	MIC	195	390	390	1562	12.5	/	/	6.25
	MBC	195	781	1562	1562	50	/	/	6.25
*E. aeroginese*	MIC	97	195	24	48.8	50	25	50	1.562
	MBC	390	390	97	195	/	100	50	1.562
*P. smartii*	MIC	48	48	97	12	6.25	100	50	0.781
	MBC	195	390	390	48	100	/	50	0.781
**Yeasts**									
*C. albicans*	MIC	3125	1562	3125	/	/	/	/	3.125
	MFC	12500	6250	6250	/	/	/	/	3.125
*C. guiliermondii*	MIC	3125	781	781	3125	/	/	/	6.25
	MFC	12500	6250	6250	/	/	/	/	12.5
*C. neoformans*	MIC	1562	195	97	3125	/	/	/	1.562
	MFC	6250	390	390	/	/	/	/	3.125
*C. luciteniae*	MIC	3125	195	195	3125	/	/	/	3.125
	MFC	12500	390	781	/	/	/	/	6.25
*C. parapsilosis*	MIC	6250	781	781	/	/	/	/	3.125
	MFC	12500	6250	6250	/	/	/	/	3.125
**Dermatophytes**									
*T. equinum*	MIC	12500	390	390	/	/	/	/	6.25
	MFC	/	1562	3125	/	/	/	/	12.5
*M. audouinii*	MIC	6250	390	781	/	/	/	/	6.25
	MFC	12500	1562	3125	/	/	/	/	6.25
*T. mentagrophytes*	MIC	/	781	781	/	/	/	/	3.125
	MFC	/	3125	3125	/	/	/	/	6.25
*M. gypseum*	MIC	12500	390	781	/	/	/	/	6.25
	MFC	/	1562	3125	/	/	/	/	12.5
*E. flocosum*	MIC	12500	390	781	/	/	/	/	3.125
	MFC	/	1562	3125	/	/	/	/	6.25

Extract^a^: leaf extract; H_f_: Hexane fraction; A_f_: Acetone fraction; MR_f_; Methanol residue fraction; RD: reference drug (Gentamycin, Nystatine and Griseofulvin for bacteria, yeasts and dermatophytes respectively); /: not active (at 12500 μg/ml for extract and fractions, and 100 μg/ml for compounds).

**Table 2:** **Minimum inhibitory and cidal concentrations (μg/ml) of the extract, fractions and compounds from the stem of** ***P pinnata*** **against bacteria and fungi**

Microorganism						Test substance				
		Extract ^b^	PE _f_	EA _f_	AR _f_	4	5	6	7	RD
**Bacteria**										
*S. aureus*	MIC	781	1562	195	390	25	1.562	6.25	3.125	0.781
	MBC	781	3125	390	1562	100	1.562	6.25	6.25	3.125
*P. aeruginosa*	MIC	781	781	390	781	100	3.125	50	6.25	0.781
	MBC	1562	3125	781	1562	/	6.25	100	12.5	1.562
*K. pneumonia*	MIC	781	781	390	781	25	6.25	12.5	6.25	6.25
	MBC	781	3125	781	1562	25	6.25	12.5	6.25	6.25
*E. coli*	MIC	24	781	97	195	25	0.781	3.125	3.125	0.781
	MBC	390	781	97	195	25	0.781	6.25	6.25	0.781
*E. faecalis*	MIC	390	3125	781	1562	/	6.25	12.5	25	6.25
	MBC	3125	6250	1562	1562	/	6.25	12.5	100	6.25
*E. aeroginese*	MIC	390	390	97	195	25	0.781	6.25	12.5	1.562
	MBC	781	781	195	390	50	1.562	12.5	25	1.562
*P. smartii*	MIC	97	390	48.8	390	6.25	0.781	3.125	1.562	0.781
	MBC	195	1562	195	781	12.5	0. 781	6.25	3.125	0.781
**Yeasts**										
*C. albicans*	MIC	/	1562	781	3125	/	/	3.125	3.125	3.125
	MFC	/	3125	781	3125	/	/	6.25	6.25	3.125
*C. guiliermondii*	MIC	12500	1562	195	3125	/	/	3.125	1.5625	6.25
	MFC	12500	6250	195	6250	/	/	12.5	1.5625	12.5
*C. neoformans*	MIC	3125	3125	195	3125	/	/	3.125	1.5625	1.562
	MFC	6250	3125	390	3125	/	/	12.5	6.25	3.125
*C. luciteniae*	MIC	3125	1562	97.7	781	/	/	3.125	3.125	3.125
	MFC	6250	1562	195	1562	/	/	3.125	6.25	6.25
*C. parapsilosis*	MIC	6250	1562	390	1562	/	/	6.25	6.25	3.125
	MFC	12500	3125	390	1562	/	/	25	12.5	3.125
**Dermatophytes**										
*T. equinum*	MIC	12500	390	390	3125	50	50	25	50	6.25
	MFC	/	1562	390	3125	100	100	25	50	12.5
*M. audouinii*	MIC	12500	781	390	3125	25	/	6.25	12.5	6.25
	MFC	/	1562	781	/	25	/	12.5	25	6.25
*T. mentagrophytes*	MIC	12500	390	390	781	25	50	25	50	3.125
	MFC	/	390	781	1562	50	/	25	50	6.25
*M. gypseum*	MIC	12500	390	195	781	12.5	25	6.25	25	6.25
	MFC	12500	1562	195	781	50	25	25	25	12.5
*E. flocosum*	MIC	12500	390	195	781	25	/	12.5	50	3.125
	MFC	12500	781	390	3125	25	/	25	100	6.25

Extract^b^: stem extract; PE_f_: Petroleum ether fraction; EA_f_: ethylacetate fraction; AR_f_; aqueous residue fraction; RD: reference drug (Gentamycin, Nystatine and Griseofulvin for bacteria, yeasts and dermatophytes respectively); /: not active (at 12500 μg/ml for extract and fractions, and 100 μg/ml for compounds).

In general, the antifungal activities of isolated compounds were relatively lower than their antibacterial activities. However **7** exhibited significant anti-yeast activity (MIC value range of 0.78-6.25 μg/ml), while **6** exhibited the best anti-dermatophytic activity (MIC value range of 6.25-25 μg/ml). Compounds **6** and **7** showed a wide spectrum of action on all the three types of microorganisms tested. Compounds **1**, **3** and **5**–**7** are isolated from *P. pinnata* for the first time and the antimicrobial activities of **5**–**7** are reported herein for the first time. Though the best antibacterial compound, **5** did not show good antifungal activities. The structure-activity relationship of compound **5** and its analogue **6**, shows that the introduction of an -OH group at C-16 in compound **6** considerably reduced its antibacterial activity, and increased its antifungal activities against the above mentioned microbial strains. The structures of **5** and **7** are similar, **7** showed more antifungal activity compared to **5** against the tested fungal species. This suggests that the introduction of a *β*-D-galactopyranose group at C-3*′* of the sugar moiety of C-3 increased the antifungal activity. In addition, comparing the MIC and MMC values of compounds **2** and **4,** it is seen that the presence of the C-3*- β*-D-glucopyranose group in **4** considerably increased the antimicrobial activity of the latter. Thus, the presence or absence of C-16-OH, C-3*′-* or C-3*-β*-D-hexopyranose groups play a critical role in determining the specific antimicrobial property of these types of oleanane triterpenoids and steroidal terpenes. The ratio MMC/MIC of compounds **4**–**7** was generally ≤ 4 with respect to all the microorganisms studied, indicative of a possible bactericidal nature of these compounds [29]. Moreso, the activities of compounds **6** and **7** were comparable to or better than the reference antibiotics on a considerable number of the tested microorganisms.

## Conclusion

The results of the present findings could be considered interesting, taking into account the global disease burden of these susceptible microorganisms, in conjunction with the search for alternative and complementary medicines. They also show that an antifungal formulation could henceforth be envisaged from the stems of *P. pinnata*.
